# Supplementary material for: Retention of indoxyl sulfate in different genotypes of ABCC2 may explain variation in tacrolimus pharmacokinetics
Source: PeerJ. 2024 Dec 18;12:e18729. doi: 10.7717/peerj.18729 (PMC11662891; doi:10.7717/peerj.18729)
Supplement: Supplemental Information 2 [file peerj-12-18729-s002.docx]

| characteristics | rs2032582(G/T/A) | | P | rs1045642(C/T) | | P | rs717620(C/T) | | P | rs2273697(G/A) | | P | rs3740066 | | P |
| --- | --- | --- | --- | --- | --- | --- | --- | --- | --- | --- | --- | --- | --- | --- | --- |
|  | GG | GA+GT+AT+TT |  | CC | CT+TT |  | CC | CT+TT |  | GG | GA+AA |  | CC | CT+TT |  |
| Age, year | 49.5(12.5) | 55.2(10.8) | 0.0175 | 50.7(12.9) | 55.8(9.9) | 0.0200 | 52.7(11.5) | 55.7(11.2) | 0.159 | 53.3(11.4) | 55.5(11.4) | 0.366 | 53.1(11.3) | 54.7(11.7) | 0.467 |
| Gender,  male (%) | 16(51.6%) | 62(66.7%) | 0.133 | 30(60.0%) | 48(64.9%) | 0.582 | 51(63.8%) | 27(61.4%) | 0.792 | 65(67%) | 13(48.1%) | 0.073 | 53(66.3%) | 25(58.1%) | 0.373 |
| BMI, kg/m2 | 21.6(3.9) | 22.4(2.7) | 0.304 | 21.8(3.4) | 22.4(2.8) | 0.314 | 22.4(3.0) | 21.8(3.1) | 0.279 | 22.3(3.0) | 21.9(3.3) | 0.576 | 22.4(3.0) | 21.9(3.1) | 0.417 |
| POD, day | 1169(719-1533) | 1329 (857-1822) | 0.258 | 1167.5 (719-1527) | 1366 (1014-1888) | 0.0396 | 1321(742-1653.5) | 1329.5(1059-1668.5) | 0.449 | 1323 (834-1638) | 1217 (756-1669) | 0.741 | 1297.5(742-1624) | 1362(1069-1963) | 0.173 |
| Diabetes, n (%) | 2(6.5%) | 10(10.8%) | 0.729 | 3(6.0%) | 9(12.2%) | 0.358 | 8(10.0%) | 4(9.1%) | 1.000 | 10(10.3%) | 2(7.4%) | 1.000 | 8(10.0%) | 4(9.3%) | 1.000 |
| Hypertension, n (%) | 3(9.7%) | 8(8.6%) | 1.000 | 3(6.0%) | 8(10.8%) | 0.523 | 6(7.5%) | 5(11.4%) | 0.469 | 9(9.3%) | 2(7.4%) | 1.000 | 6(7.5%) | 5(11.6%) | 0.444 |
| ALT, IU/L | 17.5(10.3-30.8) | 16(12.7-26.3) | 0.883 | 16.15(11.2-28.3) | 16.15(12.8-27.1) | 0.539 | 16.2(11.9-29.3) | 16.2(13.2-23.1) | 0.913 | 16.3(12.7-30.8) | 16(11-25.1) | 0.313 | 16.1(11.9-28.1) | 16.6(13.1-30.8) | 0.537 |
| AST, IU/L | 21.7(17-25.8) | 21.3(17.6-28.1) | 0.740 | 20.8(16-25.1) | 22.5(18.3-31.7) | 0.0437 | 21.8(17.6-28.9) | 21.2(17-26.4) | 0.527 | 21.1(17.5-28.8) | 23.2(17.5-25.2) | 0.969 | 21.4(17.5-27.4) | 21.7(17-28.1) | 0.979 |
| TBIL, µmol/mL | 15(10.5-21.4) | 14.1(11.1-19.2) | 0.269 | 14.95(10.5-19) | 14.3(11.1-19.6) | 0.737 | 15.2(10.4-19.9) | 13.4(11.3-17.7) | 0.317 | 14.9(11.5-19.6) | 14.2(9.8-16.8) | 0.354 | 15.1(10.8-19.6) | 13.9(10.5-18.8) | 0.526 |
| Albumin, µmol/mL | 44(41.3-45.2) | 44.7(42.3-46.3) | 0.154 | 44.1(41.7-46.3) | 44.6(42.3-46.1) | 0.531 | 44.2(41.4-46.2) | 44.5(42.3-46.2) | 0.567 | 44.3(42.2-46.3) | 44(41.3-45.2) | 0.231 | 44.4(41.6-46.3) | 43.8(42.3-46) | 0.803 |
| Uric acid, μ mol/L | 397.4(131.3) | 371(323-443) | 0.414 | 390.4(119.4) | 370.5(323-446) | 0.706 | 392.6(99.2) | 363(317-469.5) | 0.596 | 395.8(114.6) | 372(338-420) | 0.525 | 392.6(98.3) | 366(315-471) | 0.734 |
| C-reactive protein, mg/L | 2.5(1.8-3.6) | 2.6(1.9-3.8) | 0.986 | 2.45(1.8-3.2) | 2.65(2-3.9) | 0.439 | 2.7(1.9-3.8) | 2.4(1.9-3.5) | 0.512 | 2.6(1.9-3.8) | 2.5(1.9-3.6) | 0.751 | 2.7(1.9-3.8) | 2.4(1.9-3.9) | 0.671 |
| Creatine, μmol/L | 85(66-102) | 81(66-100) | 0.890 | 86(68-102) | 80.5(66-100) | 0.482 | 79.5(66-98) | 82.5(70.5-104) | 0.369 | 85(68-102) | 77.0(22.7) | 0.0648 | 81(66-98) | 81(58-104) | 0.855 |
| eGFR, mL/min/1.73 m2 | 84.3(36.8) | 87.2(31.4) | 0.672 | 83.8(35.5) | 88.3(30.8) | 0.460 | 89.4(31.8) | 81.3(34.0) | 0.191 | 85.4(34.5) | 90.6(25.2) | 0.469 | 87.4(29.8) | 85.3(38.0) | 0.728 |
| TAC concentration, ng/mL | 5.2(2.1) | 5.2(2.0) | 0.959 | 5.4(2.1) | 5.0(2.0) | 0.229 | 5.3(2.0) | 4.9(3.7-5.8) | 0.153 | 5.1(2.1) | 5.6(1.9) | 0.253 | 5.4(2.1) | 4.6(3.3-5.7) | 0.035 |
| TAC dose, mg/day | 1.6(1.0) | 1.7(0.7) | 0.872 | 1.7(0.9) | 1.6(0.7) | 0.445 | 1.7(0.9) | 1.6(0.7) | 0.895 | 1.6(0.8) | 1.9(0.9) | 0.105 | 1.7(0.9) | 1.6(0.7) | 0.946 |
| TAC concentration (ng/mL)/dose (mg/day) | 1.6(1.2-2.5) | 1.4(1.0-2.3) | 0.304 | 1.6(1.2-2.4) | 1.5(1.1-2.5) | 0.460 | 1.6(1.2-2.4) | 1.4(1.1-2.3) | 0.334 | 1.5(1.2-2.5) | 1.6(1.0-2.4) | 0.741 | 1.6(1.2-2.5) | 1.4(1.0-2.3) | 0.198 |
| IS, µg/mL | 0.7(0.4-1.3) | 0.8(0.4-1.4) | 0.729 | 0.7(0.4-1.3) | 0.8(0.4-1.4) | 0.762 | 0.6(0.3-1.4) | 0.9(0.6-1.3) | 0.0399 | 0.8(0.4-1.4) | 0.6(0.3-1.1) | 0.195 | 0.6(0.3-1.4) | 0.9(0.5-1.3) | 0.120 |
| HA, µg/mL | 0.1(0.07-0.3) | 0.2(0.08-0.5) | 0.235 | 0.1(0.06-0.4) | 0.2(0.1-0.6) | 0.0938 | 0.2(0.08-0.5) | 0.2(0.07-0.6) | 0.569 | 0.2(0.08-0.5) | 0.1(0.07-0.3) | 0.0971 | 0.2(0.07-0.4) | 0.2(0.07-0.7) | 0.247 |
